# Challenges in the Diagnosis and Management of Acquired Nontraumatic Urethral Strictures in Boys in Yaoundé, Cameroon

**DOI:** 10.1155/2016/2586458

**Published:** 2016-04-30

**Authors:** F. F. Mouafo Tambo, G. Fossi kamga, C. Kamadjou, L. Mbouche, A. S. Nwaha Makon, J. Birraux, O. G. Andze, F. F. Angwafo, P. Y. Mure

**Affiliations:** ^1^Pediatric Surgery Service, Yaoundé Gynaeco-Obstetric and Pediatric Hospital (YGOPH), Yaoundé, Cameroon; ^2^University of Geneva Teaching Hospital, Genève, Switzerland; ^3^Lyon Teaching Hospital, Lyon, France

## Abstract

*Introduction*. Urethral strictures in boys denote narrowing of the urethra which can be congenital or acquired. In case of acquired strictures, the etiology is iatrogenic or traumatic and rarely infectious or inflammatory. The aim of this study was to highlight the diagnostic and therapeutic difficulties of acquired nontraumatic urethral strictures in boys in Yaoundé, Cameroon.* Methodology*. The authors report five cases of nontraumatic urethral strictures managed at the Pediatric Surgery Department of the YGOPH over a two-year period (November 2012–November 2014). In order to confirm the diagnosis of urethral stricture, all patients were assessed with both cystourethrography and urethrocystoscopy.* Results*. In all the cases the urethra was inflammatory with either a single or multiple strictures. The surgical management included internal urethrotomy (*n* = 1), urethral dilatation (*n* = 1), vesicostomy (*n* = 2), and urethral catheterization (*n* = 3). With a median follow-up of 8.2 months (4–16 months) all patients remained symptoms-free.* Conclusion*. The authors report the difficulties encountered in the diagnosis and management of nontraumatic urethral strictures in boys at a tertiary hospital in Yaoundé, Cameroon. The existence of an inflammatory etiology of urethral strictures in boys deserves to be considered.

## 1. Introduction

Urethral strictures in male children denote narrowing of the lumen of the urethra which can be of either congenital or acquired origin [1]. When their origin is congenital, it is essentially due to posterior urethral valves or meatal stenosis. In most cases, it is an acquired disorder whose etiology may be iatrogenic or traumatic and less commonly infectious and inflammatory [2]. Hence the record of five consecutive cases of acquired nontraumatic urethral strictures in male children at the Pediatric Surgery Service of the Yaoundé Gynaeco-Obstetric and Pediatric Hospital led us to reporting our experience in the management of this condition in our setting. To the best of our knowledge, no previous report on the subject has been carried out in our milieu. The aim of this study was to emphasize the diagnostic and therapeutic difficulties in the management of these patients particularly in suboptimal conditions.

## 2. Case Presentation

### 2.1. Case  1

**Figure 1:**
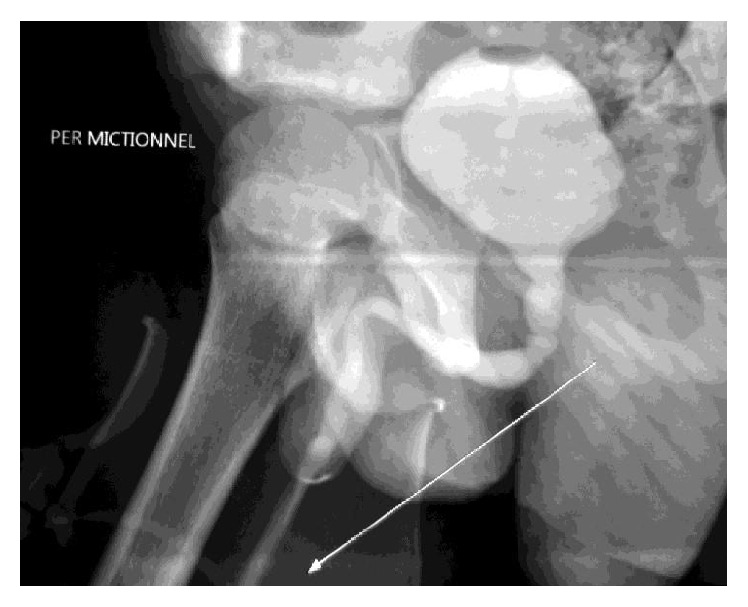
Stricture of the bulbous urethra as shown on the miction cystourethrogram (Case 1).

D. C., an 8-year-old male, was referred from the Yaoundé Central Hospital for the management of posterior urethral valves. The history revealed the onset of symptoms goes back to about a year before with obstructive voiding symptoms (straining to urinate and urinary intermittency). This prompted the diagnosis of a urinary tract infection for which he was treated with parenteral antibiotics without any improvement. An intravenous urography done thereafter was consistent with posterior urethral valves motivating admission. His past history was noncontributive, without any history of penile trauma. Circumcision had been performed during the neonatal period without any complications. Balanitis xerotica obliterans was not observed before the circumcision. On admission his physical examination was normal, except for suprapubic tenderness and a painful micturition test with a weak urinary stream. Our presumptive diagnosis was meatal stenosis or posterior urethral valves. Imaging investigations included an ultrasonography of the urinary tract that did not show any sign of urethral stenosis and a retrograde urethrocystography highlighted a stenosed anterior bulbomembranous urethra but no tight stricture (Figure 1). The urinalysis and urine culture were negative, but there were signs of inflammation including a raised erythrocyte sedimentation rate (40 mm and 80 mm at the 1st and 2nd hour, resp.). The patient was assessed with cystourethroscopy which revealed the absence of posterior urethral valves and severe inflammation of the anterior urethra close to the verumontanum and the bladder neck. A Foley catheter was left in place for 3 weeks. After a follow-up of 5 months, the patient was free of symptoms with a good urinary stream.

### 2.2. Case  2

**Figure 2:**
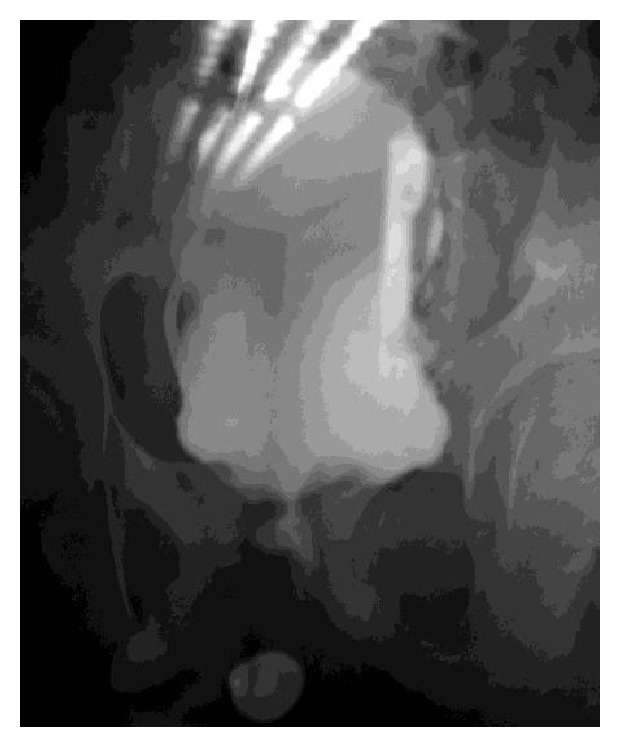
Multiple strictures of the penile and posterior urethra shown on an AP view (Case 2).

K. T., aged 13 years, was admitted to the Emergency Department of Yaoundé Gynaeco-Obstetrics and Pediatric Hospital for acute urinary retention. The history revealed that the onset of symptoms dated back to a year before; he presented with lower urinary tract symptoms and overflow incontinence. His medical history revealed a history of mental retardation (probably related to fragile X mental retardation syndrome) but he had never been a subject to any genitourinary trauma nor urethral instrumentation. Circumcision had been performed during the neonatal period without any complications. Balanitis xerotica obliterans was not observed before the circumcision. On entry, the patient presented with mild fever (37.8°C) and especially a painful convex median suprapubic mass consistent with a fully distended bladder. There was no blood in the urine. The rest of his physical examination was normal. A presumptive diagnosis of urethral calculi was evoked. Initial management included suprapubic catheterization after failure of urethral catheterization. The urinary tract ultrasound did not visualize any calculi but revealed ureterohydronephrosis with enlarged kidneys (126 mm right and 129 mm left) and a thickened irregular bladder wall. The urine culture was positive for* Klebsiella pneumoniae*, sensitive to amikacin. The erythrocyte sedimentation rate was raised (100 mm at the 1st hour and 120 mm at the 2nd hour). The kidney function tests were within normal limits. The voiding cystourethrogram done after ruling out the urinary tract infection demonstrated multiple strictures of the penile urethra, a stricture of the posterior urethra, and a flaccid bladder (Figure 2). The decision to perform an internal urethrotomy was taken after evaluating the patient with urethrocystoscopy, during which urethral dilatation was done up to the caliber of a Ch 14 Foley catheter; the latter was then introduced and was left in place for 1 month. Prophylactic antibiotherapy with oral trimethoprim-sulfamethoxazole (TMP/SMX) was started after surgery; the postoperative course was uneventful. Four months after, the outcome was favorable, and the patient was free of symptoms with a good urinary stream.

### 2.3. Case  3

**Figure 3:**
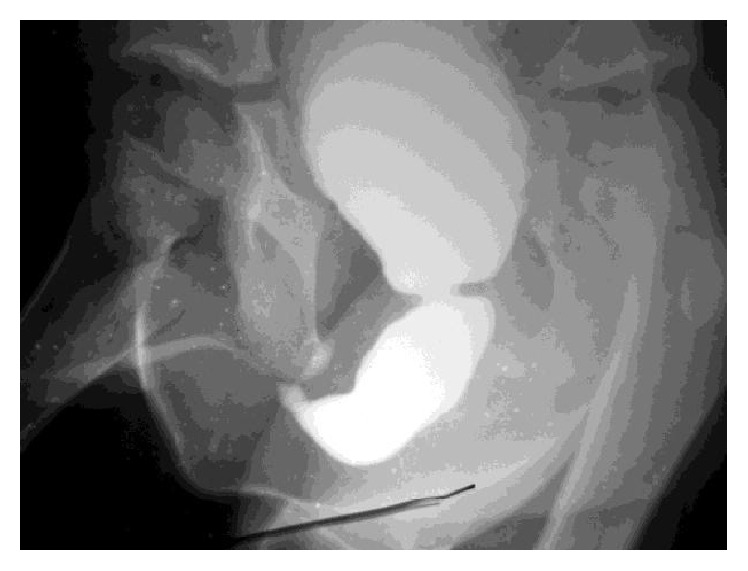
Stricture of the posterior urethra as shown on the lateral view of the MCUG (Case 3).

**Figure 4:**
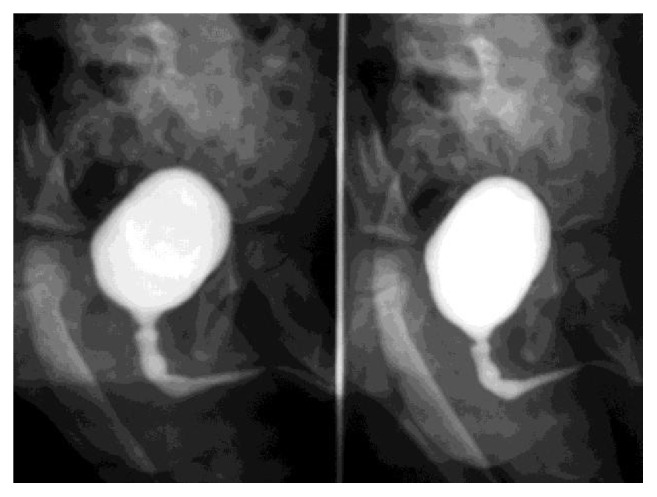
Multiple strictures of the urethra with a dilated bladder shown on MCUG (Case 3).

**Figure 5:**
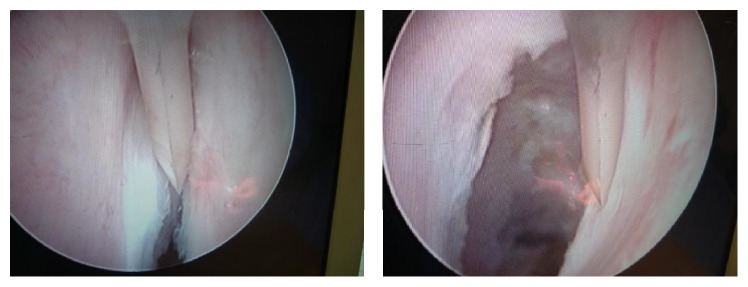
Endoscopic view of an inflammatory urethra (Case 3).

B. A., at the age 5 of years, experienced pain and difficulty on voiding. The mother reported previous similar symptoms at 8 months of age. The diagnosis of urethral meatal stricture was made. A meatotomy was performed at 2 years. The patient continued to have symptoms and presented with persistent daytime incontinence at our outpatient department. The mother denied any history of trauma but he did have a decreased force of stream and urinary terminal dribbling. Routine circumcision had been carried out during neonatal period without any complications. Lichen sclerosus was not reported before circumcision. Initial evaluation on admission revealed a palpable distended bladder and absence of urine stream. This clinical presentation was highly suggestive of urethral stricture. Ultrasonography of the lower urinary tract showed a distended bladder with an irregular thickened wall. Ultrasound studies of the upper urinary tract were normal and calcium stones were identified in the proximal urethra. A cystourethrogram demonstrated a pear shaped bladder and irregularity of the urethra: dilatation proximal and distal to two urethral strictures (Figures 3 and 4). Renal function tests were normal and urine culture was sterile despite the positive laboratory inflammation parameters (raised erythrocyte sedimentation rate: 40 mm and 80 mm at the 1st and 2nd hour, resp., and also mild hypochromic microcytic anemia). At the endoscopic evaluation tight mid-penile and posterior strictures were visualized (Figure 5). The proximal penile stricture was freely excised using the panendoscope but the posterior stricture was extremely tight. The decision to perform a vesicostomy was thus taken. Following revision, the cystoscopic examination showed periurethral scar tissue; the urethra was patent up to a size 12 Foley catheter. A catheter was left in dwelling for 4 weeks and the vesicostomy was closed. Sixteen months later, the child voided easily and his stream remained excellent.

### 2.4. Case  4

F. I., a 14-year-old teenager, presented with symptoms of painful urination, dribbling, and difficulty starting his urinary stream. The onset of symptoms had been gradual over the past 4 years. He gave no history of pyelonephritis, chills, fever, nor previous trauma. Routine circumcision had been carried out previously without any complications. Lichen sclerosus was not reported before circumcision. At the age of 10 years he experienced difficulty in voiding and acute urinary retention that requires suprapubic drainage. The diagnosis of urethral meatal stricture was made. Four years prior to admission, meatal reconstruction was realized. The biologic markers of inflammation were positive; urine studies and renal function were normal. Under general anesthesia, endoscopic evaluations demonstrated a hypertrophied bladder and a diffuse inflammation of the urethra and confirmed the presence of a stricture at the bulbous region of the urethra. The stricture area was dilated using the endoscope and a hinge 12 Foley catheter was left indwelling for 8 days. Following removal of the catheter, the patient voided normally and 12 months postoperatively, the patient has no symptoms and voids with a good stream.

### 2.5. Case  5

E. D., at the age of 07 years, presented with haematuria, frequency, difficulty in voiding, and overflow incontinence. Three weeks prior to admission, he experienced dribbling and difficult urination. The mother reported previous difficulty from infancy and recurrent urinary tract infections; she denied any fever or chills. Ultrasound studies of the lower urinary tract showed a distended thick-walled bladder and the patient was admitted at the Pediatric Surgery Service for evaluation. Routine circumcision had been carried out previously without any complications. Lichen sclerosus was not reported before circumcision. On admission, the patient presented with a good general condition. Vital signs were normal: weight: 48 kg and temperature: 37°C; the rest of the physical exam was nonsuggestive. A retrograde cystourethrogram demonstrated stricture of the posterior urethra with a diverticulum of the bladder. The etiology of this stricture was not clear; the posterior location could presume posterior urethral valves as well as an infectious stricture. The urine was sterile and renal function was normal despite the raised markers of inflammation. A vesicostomy was first performed and the postoperative course was uneventful. At cystoscopic evaluation three months later, the urethra was patent and accepted a 16 F sound. A left lateral diverticulum of the bladder probably due to obstruction was seen. This was treated by placement of an indwelling catheter and closure of the vesicostomy was done during the same procedure. The catheter was removed 5 days later and the patient was discharged. At the four-month postoperative review, his urine stream was normal and urine culture negative.

## 3. Discussion

Diagnostic difficulties in the reported cases focused on the origin of the urethral stenosis as the latter determines the management. The causes of the male urethra strictures are, indeed, a major issue in pediatric urology as they condition the treatment. The most common causes of acquired urethral strictures today are traumatic or iatrogenic [2]. In our cases, the cause was neither posterior urethra valves nor meatal stenosis after clinical and radiological assessment. The average age of 9.4 years in our patients at the time of consultation suggests already acquired voluntary bladder control and constitutes an argument against congenital etiology. Moreover, congenital urethral strictures are rare [3] and in the vast majority of cases are caused by posterior urethral valves in newborn males. The urethral stenosis is thus most likely acquired in the five cases reported. Similarly, posttraumatic strictures were ruled out since no previous injury to the urethra had been reported by the patients and their families prior to the onset of symptoms. However, the iatrogenic origin may be suspected in cases 2 and 4 given previous urethral catheterization but the topography of the lesions makes it less likely. Instrumentation or operative procedures may cause strictures located distally to the normal physiologically narrowed zones of the urethra [4]. It may as well be an acute inflammatory thrust after a prior trauma to the urethra that went unnoticed. The infectious etiology does not really fit in our series, as the urine culture was negative in most of the patients (*n* = 4). Moreover, infectious urethral strictures are secondary typically to urethritis caused by sexually transmitted infections, which remain uncommon in children. In case 2 where* Klebsiella pneumoniae* was cultured, it is presumably an acquired infectious urethral stricture. It is left to consider the inflammatory etiology, against which we found no arguments. Indeed, in all the cases reported, the biologic markers of inflammation were positive (average ESR > 40 mm and > 80 mm at the 1st and 2nd hour) and in all the cases cystoscopy demonstrated inflammation of all or part of the urethra. In addition the postoperative course after a median follow-up of 8.2 months easily fits into this etiology. In the authors' opinion, the inflammatory origin may account for a urethral stricture disease in our midst milieu. Regarding management, temporary diversion of urine specifically by vesicostomy as in cases 3 and 5 was justified to protect the bladder and upper urinary tract. Also, endoscopy is not readily available in our context even for emergency procedure. It often depends on international cooperation as was the case in our experience. Inflammatory lesions of the urethra in our patients were managed with noninvasive procedures to avoid more damage to the urethra. Resection and anastomosis was not performed in any of our cases.

## 4. Conclusion

Nontraumatic acquired strictures of the male urethra pose difficult diagnostic and therapeutic problems in our setting. There seem to be more arguments in favor of an inflammatory origin. The findings pointing to this origin will need a larger sample size to be confirmed. In the absence of urethrocystoscopy the vesicostomy remains the best option to prevent damage of the upper urinary tract.

## References

[1] Scherz H. C., Kaplan G. W. (1990). Etiology, diagnosis, and management of urethral strictures in children. *Urologic Clinics of North America*.

[2] Gibbons M. D., Koontz W. W., Smith M. J. V. (1979). Urethral strictures in boys. *Journal of Urology*.

[3] Culty T., Ravery V., Boccon-Gibod L. (2007). Les sténoses post-traumatiques de I’urètre : à propos de 105 cas. *Progrès en Urologie*.

[4] Frank J. D., Pocock R. D., Stower M. J. (1988). Urethral strictures in childhood. *British Journal of Urology*.

